# Biological Functions of Prokaryotic Amyloids in Interspecies Interactions: Facts and Assumptions

**DOI:** 10.3390/ijms21197240

**Published:** 2020-09-30

**Authors:** Anastasiia O. Kosolapova, Kirill S. Antonets, Mikhail V. Belousov, Anton A. Nizhnikov

**Affiliations:** 1 Laboratory for Proteomics of Supra-Organismal Systems, All-Russia Research Institute for Agricultural Microbiology (ARRIAM), 196608 St. Petersburg, Russiak.antonets@arriam.ru (K.S.A.); belousovmix@gmail.com (M.V.B.); 2 Faculty of Biology, St. Petersburg State University (SPbSU), 199034 St. Petersburg, Russia

**Keywords:** amyloid, bacteria, interspecies interactions, host–pathogen, host–symbiont, microbial community, outer membrane protein, biofilm, Omp, toxin

## Abstract

Amyloids are fibrillar protein aggregates with an ordered spatial structure called “cross-β”. While some amyloids are associated with development of approximately 50 incurable diseases of humans and animals, the others perform various crucial physiological functions. The greatest diversity of amyloids functions is identified within prokaryotic species where they, being the components of the biofilm matrix, function as adhesins, regulate the activity of toxins and virulence factors, and compose extracellular protein layers. Amyloid state is widely used by different pathogenic bacterial species in their interactions with eukaryotic organisms. These amyloids, being functional for bacteria that produce them, are associated with various bacterial infections in humans and animals. Thus, the repertoire of the disease-associated amyloids includes not only dozens of pathological amyloids of mammalian origin but also numerous microbial amyloids. Although the ability of symbiotic microorganisms to produce amyloids has recently been demonstrated, functional roles of prokaryotic amyloids in host–symbiont interactions as well as in the interspecies interactions within the prokaryotic communities remain poorly studied. Here, we summarize the current findings in the field of prokaryotic amyloids, classify different interspecies interactions where these amyloids are involved, and hypothesize about their real occurrence in nature as well as their roles in pathogenesis and symbiosis.

## 1. Introduction

The term “amyloid” dates back to the previous century. In 1838, Matthias Schleiden introduced “amyloid” (from Latin “amylum”—starch) to describe a starch material in plant cells [1]. In 1854, Rudolf Virchow first used “amyloid” to characterize cerebral inclusions that colored blue in a reaction with iodine [2]. Based on the iodine test reaction, Virchow hypothesized about polysaccharide nature of pathological inclusions in so-called “waxy” human organs that underwent irreversible changes called amyloidosis [1]. Five years later, in 1859, August Kekulé and Carl Friedreich showed that the inclusions in “waxy” spleen were enriched in nitrogen and were rather proteinaceous than starchy [3].

Currently, the term “amyloid” refers to the highly ordered protein aggregates formed by unbranched fibrils the protein monomers of which are stacked by intermolecular β-sheets [4] composed of β-strands running perpendicular to the fibril axis and connected via hydrogen bonds [5]. The spatial organization of amyloid fibrils determines “cross-β” diffraction pattern characterized by two scattering diffraction signals: ~4.7 Å, corresponding to the interstrand distance, and ~10 Å, corresponding to the distance between β-sheets [6,7]. This structure is typical of amyloids, yet there is no rule without exception: phenol-soluble modulin α3 (PSMα3), a secreted protein of *Staphylococcus aureus*, forms fibrils possessing physicochemical properties of amyloids but shows “cross-α” diffraction pattern that corresponds to perpendicularly stacked α-helices rather than to β-sheets in “cross-β” structure [8].

Unique physicochemical properties of amyloid fibrils allow discriminating amyloids from the non-amyloid protein aggregates. Two defining features of amyloids are: (i) their ability to form mainly unbranched fibrils that can be shown with electron or atomic force microscopy [9,10]; and (ii) their “cross-β” structure, which can be directly demonstrated by X-ray diffraction (XRD) [6] or solid state nuclear magnetic resonance (SS-NMR) [11]. Circular dichroism spectroscopy (CD), often used to evaluate the enrichment of protein aggregates in β-sheets, cannot prove their “cross-β” structure [12]. Amyloids are resistant to treatment with ionic detergents such as sodium dodecyl sulfate (SDS) [13] and proteases [14], although these properties vary significantly depending on the amyloids themselves and the proteins from which they are formed [15,16]. Amyloids also bind specific dyes [17], which was demonstrated for the first time in 1922 with Congo red (CR) dye [18]. Further, amyloids have been shown to exhibit “apple-green” birefringence in polarized light upon CR binding [19] and demonstrate specific fluorescence emission spectra [20]. Another widely used dye for the analysis of amyloids is Thioflavin T (ThT), which binding to amyloids leads to an increased fluorescence intensity [21].

For more than 150 years, amyloid studies were mainly focused on their roles in the development of incurable diseases in humans and animals, such as Alzheimer’s disease and various localized and systemic amyloidoses. Despite extensive study, the molecular mechanisms of amyloid toxicity remain unclear. To date, two dominant hypotheses have been proposed [22]. Amyloid hypothesis suggests that deposition of amyloid fibrils themselves causes toxicity leading to cell death [23]. The second hypothesis postulates that soluble oligomers rather than fibrillar polymers cause the main cytotoxic effect [24]. Both hypotheses are unable to fully explain observed data. Thus, there are new currently emerging hypotheses. In particular, lipid-chaperone hypothesis is based on the observation that toxicity of amyloidogenic proteins is associated with their ability to damage cell membranes and suggests that phospholipids can act as molecular chaperones promoting interaction of amyloidogenic proteins with cell membranes [22,25].

The paradigm describing amyloid as harmful agents shifted with the discovery of functional amyloids that can perform various physiological roles [26,27]. To date, functional amyloids have been identified within all three domains of life, *Archaea*, *Bacteria*, and *Eukarya*, and their current number is approximately equal or even exceeds the number of pathogenic ones [27,28]. The greatest diversity of functional amyloids is identified within prokaryotic organisms. Since the discovery of amyloid properties of the curli fimbriae of *Escherichia coli* [29], more than 30 amyloidogenic proteins of *Bacteria* and *Archaea* have been identified. Part of them form amyloid fibrils under the native conditions and act as functional amyloids involved in the interspecies interactions with their hosts or within microbial communities. The variety of the prokaryotic amyloid-forming proteins, the methods that have been used to analyze their amyloid properties in vitro and in vivo, and their functions in the amyloid and soluble states are summarized in Table 1. In this paper, we review the published scientific data on the diversity of prokaryotic amyloids and discuss their biological functions with regard to their role in different types of interspecies interactions in both pathogenic and symbiotic aspects.

## 2. Amyloids of Biofilms and Their Involvement in Host–Pathogen Interactions and Interspecies Interactions within Prokaryotic Communities

The highest number of the identified functional amyloids of prokaryotes is represented by the biofilm components. Biofilm is a community of microorganisms encapsulated in hydrated extracellular polymeric substances (EPS) [73]. EPS account for almost 90% of the dry weight of a biofilm and include polysaccharides, eDNA, lipids, and proteins [73]. The biofilm proteins include the extracellular enzymes, carrying out degradation and remodulation of EPS, and structural proteins, providing stability and integrity to biofilm [74]. The stability of amyloid fibrils, originating from their spatial structure, makes them perfect structural proteins of the biofilm EPS. Thus, amyloids, making the biofilms stable, serve as scaffolding proteins, as well as play a role in surface and intercellular adhesion [75]. At the same time, a biofilm formation is linked with the development of 65% of all bacterial infections and 80% of chronic bacterial infections [76] such as periodontitis, chronic rhinosinusitis, chronic otitis media, chronic urinary tract infections, and cystic fibrosis pneumonia [77]. Biofilm formation creates a local microenvironment (such as anaerobic conditions or zones with lowered pH), to protect the microbial community from the antibiotic treatment, host defense, and environmental stresses [78] and contributes to formation of so-called “persister” microbial sub-population formed by dormant, multi-drug resistant cells [79]. Thus, amyloids that have been identified within the pathogenic bacteria and being part and parcel of the biofilm matrix can act as virulence and pathogenesis factors [80].

The curli are the main structural proteins of EPS of *Escherichia coli* biofilms [29,31,81], adhering to both biotic and abiotic surfaces [82,83,84]. In 2002, amyloid properties were demonstrated for *E. coli* curlin CsgA [29] and in 2007 for *Salmonella enterica* curlin AgfA [31]. Curli amyloid formation involves secretion system Type VIII and is controlled by the expression of two operons—*csgABC* and *csgDEFG* (curli-specific genes)—in *E. coli* [29]. CsgA is the main structural protein while CsgB nucleates CsgA polymerization on the cell surface [85]. CsgC, the third gene from *csgABC* operon, is a periplasmic chaperone that prevents a premature CsgA polymerization [86]. Lipoprotein CsgG forms a pore in the outer membrane of bacterial cells and mediates the transport of the curli subunits to the cell surface [87]. CsgE and CsgF proteins facilitate CsgA and CsgB transport through CsgG pore [88]. CsgE interacts directly with the pore and secreted proteins and acts as a secretion adaptor [88]. The precise function of CsgF remains unclear, but it is required for the normal functioning of the CsgB nucleator [88,89].

Despite curli fimbriae were initially characterized within clinical isolates, the precise role of amyloid formation of those proteins in infection remained unclear [90,91]. Indeed, curli operons are not only present in the genomes of pathogenic strains of *Proteobacteria* but are also widespread within the non-pathogenic strains [92]; and curli homologs have also been found within *Firmicutes*, *Thermodesulfobacteria*, and *Bacteroidetes* phyla [92], including *Porphyromonas gingivalis* [93].

The curli fimbriae apparently take part in bacteria’s adhesion to host cells [94], interact with the host proteins [95,96], and trigger the host immune response [97] during an infection. The curli-producing *E. coli* and *Salmonella* spp. strains are highly adhesive to a variety of cell lines. Thus, curli-producing K-12 *E. coli* has demonstrated a higher level of adherence to human uroepithelial cells in comparison to curli deficient strains [94]. Similarly, higher levels of curli production in *S. typhimurium* SR-11 are linked to adherence to a murine small intestinal epithelial [98]. Nevertheless, Δ*csgA* strain of enteroaggregative *E. coli* (EAEC) has not shown any decrease in adherence to mammalian cells, suggesting that the *E. coli* system of adhesion to host cells includes not only the curli fimbriae but a broad repertoire of molecular factors [99]. Moreover, curli expression levels have been significantly lowered within the enterohemorrhagic *E. coli* [100,101] and invasive *Salmonella* spp. strains [102].

The curli interact with the host proteins including fibronectin, laminin, and plasminogen [90,103,104]. They also interact with Toll-like receptors, which leads to an innate immune system activation [105,106]. On the contrary, the curli can protect bacterial cells from the immune reactions via antimicrobial peptides sequestering [107] and inhibition of the classical pathway of the complement cascade activation [108].

The Gram-negative bacterium *Pseudomonas aeruginosa* is a cause of nosocomial and chronic infections associated with the biofilm formation, for example during cystic fibrosis pneumonia [109]. The biofilm matrix of *Pseudomonas* species includes amyloid fibrils formed by Fap proteins [33]. Amyloid fibril formation in *Pseudomonas* is controlled by a *fapABCDEF* operon, evolutionally distant from the curli system of *E. coli* [33]. Unlike the curli system, *fap* genes are unique for *Proteobacteria* species [110]. FapC is the main structural component of amyloid fibrils, whereas FapB, similar to CsgB from curlin system, acts as a nucleator of fibril polymerization [34]. Transport of FapB and FapC subunits to the cell surface is facilitated by FapF protein which forms trimer pores in the outer membrane of bacteria [111].

Fap amyloid fibrils increase the biofilm hydrophobicity, facilitate mechanical stiffness [112], and reversibly bind quorum sensing molecules, supporting their role as a reservoir for signal molecules that can modulate the reaction of the microbial community to turbulent environmental conditions [35]. Similar to curli, Fap proteins contribute to bacterial adhesion to a substrate. Thus, *Pseudomonas* strains overexpressing *fap* operon have a highly adhesive phenotype and an enhanced ability to form biofilms [33,34]. However, overexpression of *fap* operon notably changes the complete proteomic landscape, thus it is impossible to assume the direct connection between Fap amyloidogenesis and the altered phenotype [113]. The role of Fap proteins in *Pseudomonas* virulence has been demonstrated using *P. aureginosa* mutant strain with *fapC* deletion. Strains with *fapC* deletion had lowered virulence to *Caenorhabditis elegans* [114]. In murine models of acute and chronic infections, *fap* operon transcription in *P. aureginosa* was also significantly elevated [115].

Gram-positive bacterium *Bacillus subtilis* forms biofilms on the surface of solid agar plates and floating biofilms, or pellicles, at the air–liquid interface [116]. TasA protein, the main component of *Bacillus* biofilm EPS [117], can form amyloids both in vitro and in vivo [45,46,47]. While *B. subtilis* is a soil-dwelling non-pathogenic bacterium, *Bacillus cereus* is a soil bacterium responsible for the development of food-borne disease. However, the role of biofilm formation and TasA amyloid formation in a particular disease development is unclear. At the same time, TasA amyloids of *Bacillus* apparently contribute to the interspecies interaction in complex biofilm communities as TasA amyloid fibrils adhere to *Streptococcus mutans* exopolysaccharides during the initial steps of multispecies biofilm formation [118].

Biofilms are the main form for *Streptococcus mutans*—a Gram-positive bacterium involved in the dental plaques and cavities formation [119,120]. Within the proteins of *S. mutans* amyloid formation in biofilm, EPS has been demonstrated for adhesin P1, WapA, and Smu_63c proteins [56,57]. Adhesin P1 and WapA protein represent substrates of sortase—an enzyme cleaving the C-terminal signal motif of proteins and attaching them to the cell wall through transpeptidase reaction [121]. As a result of adhesin P1 and WapA protein cleavage amyloid-forming fragments, C123 and AgA, respectively, are generated [57]. Smu_63c is a secreted protein that forms amyloids under acid conditions. These amyloids act as negative regulators of genetic competence and biofilm cell density [57]. The deletion of one of the genes encoding amyloid-forming proteins was shown not to affect the ability of *S. mutans* to form biofilms. At the same time, double (lacking in adhesin P1 and WapA) or triple deletions lead to decreased biofilm formation [57]. Mutants lacking in the adhesin P1 gene have a lowered virulence in the murine cavity models, but the precise role of adhesin P1 amyloidogenesis in virulence is still unclear [122].

Similar to *P. aeruginosa*, *Staphylococcus* species, *S. aureus* and *S. epidermidis*, are the leading causes of nosocomial infections [123]. At the same time, *S. aureus* as well as *S. epidermidis* can act not only as pathogens but as a part of the normal skin microbiome. *Staphylococcus* biofilm formation promotes adhesion and substrate colonization, including multicellular host tissues, as well as contributes to protection against antibiotic agents and immune system elements [124]. Thus, the biochemical content of *Staphylococcus* biofilms is a target of extensive research. The extracellular polymeric substances of staphylococcal biofilms include a variety of amyloid proteins, but their role in host–pathogen interactions have not yet been elucidated.

Sbp and Aap are amyloid-forming proteins of *Staphylococcus epidermidis* [54,55]. Sbp is a small (18 kDa) extracellular protein that forms the biofilm scaffolds [125]. The amyloid properties of Sbp have been demonstrated in vitro and in *E. coli* cells [55]. Aap is a multidomain protein associated with the bacterial cell wall. Aap includes the *N*-terminal region of tandem A-repeats, L-type lectin domain, the region of tandem B-repeats, the proline/glycine-enriched domain, and the C-terminal sortase recognition motif [126]. The ability to form amyloids was demonstrated in vitro for the B-repeats domain. Amyloid formation by B-repeats domain of Aap has a Zn^2+^-dependent manner and requires metal ions for assembly. The peptides identified as B-repeats and lectin domains of Aap protein were also present in detergent-resistant aggregates from *S. epidermidis* biofilms [54]. These data are consistent with the research suggesting that Aap protein takes part in biofilm formation in a processed form, lacking the *N*-terminal domain [127,128]. Sbp and Aap colocalization in biofilms was demonstrated [125] unlike physical interaction in vitro [55].

There is a variety of the identified amyloid-forming proteins composing the extracellular biofilm matrix of *Staphylococcus aureus*. In 2012, phenol-soluble modulins (PSMs) were identified as a part of fibrils in the biofilm matrix of *S. aureus*. PSMs also form amyloid fibrils in vitro [48]. In the amyloid state, PSMs stabilize biofilms [48], whereas monomeric PSMs facilitate biofilm detachment [129]. Extracellular DNA (eDNA) is required for PSMs polymerization, so eDNA can act as a nucleator in the amyloid formation [130]. The amyloid properties have been demonstrated for the N-terminal leader peptide of ArgD propeptide (*N*-ArgD) as well. *N*-ArgD is a naturally occurring cleavage product of ArgD, appearing due to the AIP (autoinducing peptide) maturation [50] and identified as a part of fibrils, composing the biofilm matrix of *S. aureus*. The SuhB protein of *S. aureus* forms amyloids under overexpression in *E. coli* cells [49]. The precise function of SuhB remains unknown, but the *suhB* mutant strain is impaired (in terms) of biofilm formation [131]. Another *S. aureus* protein that can form amyloids extracellularly is called Bap (biofilm-associated protein) [132]. Bap is a multidomain protein anchored to the bacterial cell wall. The N-terminal domain of Bap is cleaved as a result of the Bap processing [133]. The cleaved fragment forms amyloid fibrils in the extracellular space at acidic conditions and low Ca^2+^ concentration. The Ca^2+^ concentration increase leads to acquiring a stable globular conformation of the *N*-terminal domain of Bap [51]. Thus, the *N*-terminal domain can act not only as a scaffold protein of biofilm but also as a sensor [75]. Local acidosis, the pH decrease, appears in vivo during staphylococcal infection due to glucose utilization by these microorganisms and are accompanied by the host’s inflammatory response [132]. Within *S. aureus* strains, *bap* gene has been identified within bovine mastitis isolates [133] but not within human clinical isolates. Deletion in the *bap* gene leads to a lowered capacity to adhere to the bovine epithelial cells. *S. aureus* Δ*bap* strain cell titer is also significantly lower at 10 days post-infection [51]. Notably, Esp—the Bap ortholog of *Enterococcus faecalis*, a commensal bacterium capable of inducing nosocomial infection—forms amyloids, supporting the idea of the prevalence of amyloid formation by Bap-like proteins in biofilm matrix [53].

Pathogenic bacteria can also adhere to the host tissues in a biofilm-independent way. In particular, *Mycobacterium tuberculosis* possesses adhesive structures called pili. MTP (*Mycobacterium tuberculosis* pili) are structurally similar to *E. coli* curli and able to form amyloid fibrils [69]. The *mtp* gene has been identified only within the pathogenic strains of *M. tuberculosis*, supporting the key role of MTP in mycobacterial virulence [134]. MTPs bind laminin in vitro while Δ*mtp* strain is unable to bind it [69]. Moreover, mutants show a lowered ability to adhere and invade macrophages and alveolar epithelial cells [135].

Overall, amyloids are widespread structural components of prokaryotic biofilms. Interestingly, not only bacteria but also archaea can contain amyloids in their EPS. For instance, in 2014, the *Haloferax volcanii* biofilm extracellular matrix was demonstrated to bind ThT and CR dyes with the specific fluorescence [70]. In bacterial biofilms, the amyloids form a scaffold and facilitate their stiffness and integrity. Amyloids may also contribute to intercellular and surface adhesion, which makes them one of the key virulence factors of pathogenic bacteria. Thus, the crucial role of amyloids of biofilms in adhesion is apparently widespread across various prokaryotes, thus allowing us to suppose that there are numerous still unknown biofilm-associated amyloids underlying the pathogenesis and development of infectious diseases. Considering that the number of only human pathogenic bacteria species is about 1500 [136] and 65% of them form biofilms in disease-associated processes [55], the real number of such prokaryotic amyloids involved in pathogenesis in humans and animals could exceed hundreds and even thousands. The interactions between bacteria in microbial communities represent another type of interspecies interactions where the bacterial biofilm amyloids are involved by providing the cell adhesion to heterogeneous exopolysaccharides and where the number of yet unidentified prokaryotic amyloids could be remarkably high.

## 3. Amyloids of the Outer Membrane Proteins and Their Probable Roles in Host–Pathogen and Host–Symbiont Interactions

Fibril formation exhibiting several properties of amyloids has been demonstrated for the outer membrane proteins of *Proteobacteria* and associated with their virulence. In particular, the full-length OmpA of *E. coli* and its *N*-terminal domain form ThT-binding fibrils in vitro [37]. The role of OmpA protein in the pathogenicity of *E. coli* and other bacteria is thoroughly studied so far. OmpA is believed to contribute to bacterial adhesion and invasion, as well as to the antimicrobial peptide resistance [137]. Meningitic *E. coli* strains deficient in *ompA* are less virulent and invasive in the chick embryo and rat models [138]. In uropathogenic *E. coli* OmpA contributes to the infection persistence. As Δ*ompA* strain adheres and invades the bladder epithelium, the number of formed *E. coli* colonies is lower in comparison to the wild-type strain. Moreover, *ompA* expression is increased between 16 and 20 h after infection [139]. OmpA is notably overexpressed during the biofilm formation [140]. The facts listed above allow use to suggest that OmpA amyloids could be involved in the biofilm formation and associated with virulence of *E. coli*.

OmpC, another amyloid-forming outer membrane protein of *E. coli* [38], forms fibrillar aggregates in vitro that are resistant to proteinase K treatment. OmpC fibrils stained with ThT demonstrate a specific peak of fluorescence emission as well as OmpC fibrils stained with Congo red show green birefringence under polarized light [38]. OmpC has been detected in the brain of murine models pointing to its possible role in the neurodegeneration and formation of a spongiform encephalopathy [38]. Similar to the OmpA protein, OmpC takes part in adhesion and invasion of pathogenic strains of *E. coli*. In the avian pathogenic *E. coli* strains, deletion of *ompC* has led to a drop in adherence and ability to invade the murine brain microvascular endothelial cells. The *ompC* deletion also led to a decreased colonization and invasion capacity in ducklings and murine models [141].

OmpP2-like protein of *Mannheimia haemolytica*, an outer membrane porin of *Pasteurellaceae* [142], forms extracellular fibrils in vivo [39]. After incubation, a purified OmpP2-like protein forms polymeric aggregates binding CR. OmpP2-like protein apparently is a part of the biofilm matrix. Its role in adherence of *M. haemolytica* to host cells was demonstrated using the adenocarcinomic human alveolar basal epithelial cells [39].

Outer membrane proteins of symbiotic bacteria can also form amyloid fibrils. RopA and RopB are outer membrane proteins of plant symbiotic bacterium *Rhizobium leguminosarum*, which are predicted to form pores in bacterial outer membrane [143,144]. The levels of production of RopA and RopB proteins rises at the initial steps of nodulation [145]. These data suggest that RopA and RopB proteins are required for the early stages of the plant-bacteria symbiosis. In vitro, RopA and RopB form fibrillar aggregates exhibiting typical physicochemical properties of amyloids including green birefringence in polarized light upon CR staining, binding of ThT, resistance to proteases and ionic detergents treatment. In vivo, RopA and RopB form extracellular amyloid fibrils after the prolonged incubation of *R. leguminosarum* cells on the cultural media [40]. Moreover, the amount and size of the aggregates formed by RopA protein after induction of the nodulation process in free-living culture is increased after flavonoid treatment [40]. Based on these observations, RopA and RopB hypothetically act as adhesins and represent a part of the EPS of biofilms formed by *R. leguminosarum* on different surfaces including the plant roots, thus participating in colonization of plant by bacteria cells. Considering the increasing number of the RopA amyloids after flavonoid stimulation, a more specific role of amyloids of this protein at the initial stages of plant–microbe symbiosis can also be proposed.

The proteins discussed in this section can potentially act as the outer membrane porins possessing a β-barrel channel, thus facilitating molecular transport through the outer membrane and perform additional functions such as membrane stabilization and intercellular adhesion [146]. In vivo amyloid formation mechanisms by these proteins are not fully understood and could be realized either through β-barrel to the amyloid transition or via the alternative folding ways of β-barrels and ordered amyloid aggregates [147]. The amyloids formed by outer membrane porins of *Proteobacteria* apparently contribute to both host–pathogen and host–symbiont interactions, thus drawing a line of similarity between the mechanisms of virulence of both symbiotic and pathogenic bacteria.

## 4. Amyloids of Bacterial Toxins and Their Contribution to Pathogenesis

The amyloid state is used by prokaryotes to regulate the activity of several toxins via inactivating or storing them. Microcin E492 (Mcc) is a bacteriocin of *Klebsiella pneumoniae*. In its soluble form, microcin E492 forms pores in membranes of *Enterobacter* species. The pore formation leads to membrane potential drop and, as a result, a cell death [148]. *K. pneumoniae* produces an active soluble form of microcin E492 during the exponential growth phase. At the stationary growth phase, the toxin becomes inactivated in the amyloid form [41,149]. Listeriolysin O (LLO) of *Listeria monocytogenes* is another bacterial toxin that is inactivated in the amyloid state [64]. *L. monocytogenes* is the intracellular human pathogen. Invading a cell, *L. monocytogenes* gets inside of phagolysosome. The release of the bacteria into the cytoplasm is driven by listeriolysin O activity. LLO is active in acidic conditions of the phagolysosomes and forms pores in their membrane, which leads to the release of *L. monocytogenes* [150]. The increase of pH in cytoplasm promotes the polymerization of LLO with amyloid fibrils formation and toxin inactivation [64,151].

The amyloid formation can result not only in inhibiting but also in activating of a toxin function. The harpins are Gram-negative plant pathogenic bacteria proteins. Harpins are transported via secretion system Type III to extracellular space, where these proteins of *Xanthomonas axonopodis*, *Pseudomonas syringae*, and *Erwinia amylovora* form amyloid fibrils [43]. The precise function of harpins and their amyloid fibrils remains unclear. The harpins’ secretion triggers a hypersensitive response in plants [152,153]. The hypersensitive response is a protective mechanism preventing the spread of pathogens across plant tissues due to a rapid cell death in the localized region [154]. The way harpins cause hypersensitive response is unknown, but there are pieces of evidence, proving the ability of harpins to form pores in the cell membranes [155] and promote their depolarization [156].

Thus, amyloid formation may orchestrate the prokaryotic toxins’ activity in two ways, i.e., activating them as in the case of amyloid harpins or inactivating similar to microcin E492 and listeriolysin O, which contributes to the host–pathogen interactions of bacteria with multicellular hosts and antagonistic interactions within prokaryotic communities.

## 5. Amyloids of Extracellular Protein Layers

The amyloid formation within the protein layer surrounding the cell is widespread across prokaryotes. In particular, the amyloids can form additional extracellular layers changing cell surface properties. *Actinobacteria* species have a complex life cycle that includes hyphae and spores formation. Growth of hyphae and transition to the next stage of the life cycle requires the formation of additional protein layers modulating cell surface properties including an increase in hydrophobicity [157]. Chaplins are proteins of *Streptomyces coelicolor* that form amyloids on the surface of spores and aerial hyphae [158]. Chaplins secretion on the surface of the developing hyphae increases their hydrophobicity and lowers the water surface tension [65,158]. Rodlin RdlA, another amyloid-forming protein of *S. coelicolor*, is secreted at the later stages of hyphae development [67]. Together with chaplins, rodlins form a protein coat of spores, increasing their stiffness and hydrophobicity [157].

As opposed to rodlins and chaplins amyloid-forming bioemulsifier BE-AM1 from a Gram-positive bacterium, *Solibacillus silvestris*, lowers the hydrophobic properties of the cell surface. Bioemulsifier BE-AM1 production also facilitates an intercellular adhesion and biofilm formation [63].

Amyloids are a part of protein sheaths of archaea. Thus, the main component of sheaths of methanogenic thermophilic archaeon *Methanosaeta thermophila* is amyloidogenic protein MspA [71]. Archaeal sheaths are protein envelopes that encapsulate several cells. The cell division leads to the formation of long chains of cells under the sheath [159]. The sheaths protect archaea against protists and regulate cell turgor pressure [159]. Amyloid properties of MspA of *M. thermophila* increase the stability of sheaths in an extreme environment [71].

## 6. Amyloid Formation by Cytoplasmic Prokaryotic Proteins

The proteins listed above are secreted and form the amyloids extracellularly. However, over the last few years, the list of prokaryotic amyloids was expanded with the bacterial cytoplasmic proteins. CarD is an RNA polymerase-binding transcription factor of *Mycobacterium tuberculosis* stabilizing the transcription initiation complex [160]. CarD forms fibrils that can bind ThT with fluorescence enhancement in vitro. CarD overexpression in *E. coli* cells also leads to ThS binding aggregates [68]. HelD is a helicase of *Bacillus subtilis* that also interacts with RNA polymerase. HelD forms amyloid fibrils in vitro and forms ThS binding aggregates when expressed in *E. coli* cells [59]. The ability of HelD and CarD to form amyloids in vivo, under native conditions, and a potential physiological role of that processes require further investigations. Amyloid formation by HelD and CarD can represent the mechanisms of the functional protein inactivation via amyloid formation. As both proteins interact with RNA-polymerase, their polymerization can occur due to environmental changes and alter transcriptome and phenotype of cells.

A transcriptional regulator *Rho* of *Clostridium botulinum* (Cb-Rho) stands out as exceptional as its prion-like domain is able to form not only amyloids but also a prion, a self-propagating protein aggregate [60]. The prion-like domain of Cb-Rho was demonstrated to switch conformation to the amyloid state in *E. coli* cells without overexpression and to be able to propagate in more than 100 generations. Cb-Rho Amyloid formation leads to its decreased activity and changes in genome expression. There are also data on the ability of several amyloid-forming archaeal domains to act as prions, which propose that prion formation is spread within prokaryotic organisms [161].

Thus, the role of amyloids of the extracellular protein levels and cytoplasm of prokaryotes in the interspecies interactions cannot be excluded especially given the position of interactions between different species of prokaryotes with microbial communities.

## 7. Amyloids of Prokaryotes and Interspecies Interactions: A Tip of the Iceberg

**Figure 1:**
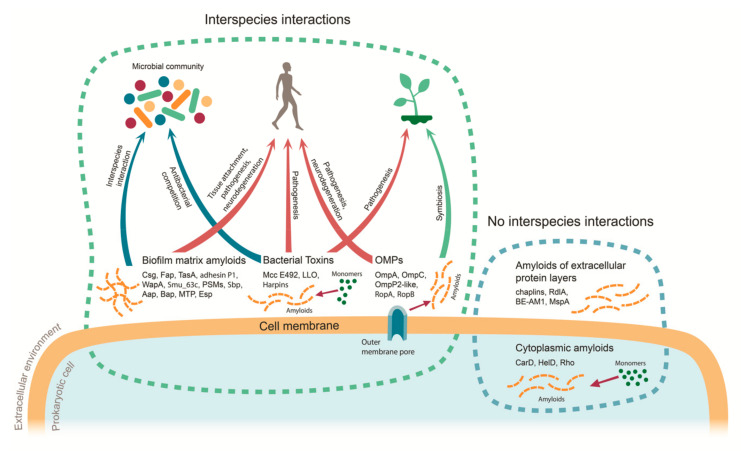
Functions of prokaryotic amyloids in the interspecies interactions. Dots denote monomeric proteins, striated lines denote amyloid fibrils, and monomer-to-amyloid conversion is shown as a thin arrow. Thick arrows represent different types of interspecies interactions: red for Type I (host–pathogen), blue for Type II (interactions within microbial community), and green for Type III (host–symbiont). MTP—*Mycobacterium tuberculosis* pili; LLO—listeriolysin O; OMPs—outer membrane proteins; PSMs—phenol-soluble modulins.

The data discussed in the previous sections and summarized in Table 1 indicate that more than 30 amyloid-forming proteins of prokaryotes have been identified to date and most of them contribute in some way to interspecies interactions. Such interactions, mediated by prokaryotic amyloids, can be divided into three types: Type I, host–pathogen interactions in which most of the identified prokaryotic amyloids are involved; Type II, antagonistic and synergistic interspecies interactions within microbial communities; and Type III, host–symbiont interactions (Figure 1 and Table 1). While the involvement of prokaryotic amyloids in the host–pathogen interactions has been scrutinized, the range of biological roles of amyloids in two latter types of interactions remains poorly studied and represents an intriguing question to be addressed in the future.

The involvement of the prokaryotic amyloids in the Type I (host–pathogen) interactions is related to the function of these amyloids or their structural proteins as the virulence factors or toxins. Most functional amyloids produced by bacterial pathogens of animals can act as virulence factors [80], predominantly as adhesins and structural proteins of the biofilm matrix, as biofilm formation is required for the multicellular host’s tissues colonization during infection. Moreover, bacterial amyloids can trigger the immune response in the host–pathogen interactions and protect the microorganisms from the host immune response [80]. Toxins represent the second subtype of functional prokaryotic amyloids involved in host–pathogen interactions. In this case, the amyloid state of a toxin can be either active (harpins) [43] or inactive (listeriolysin O [151] and microcin E492 [41]). Accumulation of bacterial toxins in the inactive amyloid state represents the storage function of amyloids (formation of dormant aggregates for further use). This function has been described not only in prokaryotes but also in eukaryotes, particularly, in animals (hormones and amyloid bodies) and plants (seed storage proteins) [162,163,164]. The existence of other functional virulence-associated amyloids mediating the host–pathogen interactions cannot be excluded. For example, evolutionary conservative domain of the mucin degrading metalloprotease YghJ involved in the virulence of enterotoxigenic *E. coli* [165] forms amyloids in vitro and being secreted to the cell surface of *E. coli* in the C-DAG system [166]. At the same time, the full-length YghJ forms detergent resistant aggregates in vivo [167].

Amyloid-forming bacterial toxins participate not only in the Type I pathogen–host interactions but also in the Type II antagonistic interspecies interactions within prokaryotic communities such as microcin E492 (see Section 4). Even though these communities seem to represent a promising source to search for novel amyloids due to the formation of biofilms, little is known about biological roles of prokaryotic amyloids in such interspecies systems because the amyloid formation of secreted proteins is mostly studied in the model single-species biofilms. Nevertheless, other examples of the amyloids involved in the interspecies interactions within prokaryotic communities have recently been revealed. In particular, amyloid fibrils of TasA of *Bacillus subtilis* bind exopolysaccharides of *Streptococcus mutans* at the initial steps of biofilm formation, thus representing an example of the amyloid involvement in the synergistic Type II interactions [118]. Several indirect pieces of evidence also suggest the involvement of the amyloid formation in the Type II interactions. Cross-seeding, the process, in which amyloids of one protein cause polymerization of another [168], between bacterial amyloid-forming proteins can contribute to the development of the multicellular biofilms. It has been shown that curli from *Escherichia coli*, *Salmonella typhimurium* LT2, and *Citrobacter koseri* are able to cross-seed each other in vitro [169]. Cross-seeding of curli has also occurred in vivo in two-species biofilms of *E. coli* and *S. typhimurium* [169]. The ability of non-homologs bacterial amyloid-forming proteins to cross-seed requires further investigation and may highlight some probable interactions between amyloids of different prokaryotic species in the future.

Microbial communities may also trigger the amyloid-associated neurodegenerative diseases development such as Alzheimer’s and Parkinson’s diseases, in their multicellular hosts (humans and animals). Thus, they demonstrate an unusual mix of Type I and II interactions though molecular mechanisms underlying these effects remain poorly understood [170]. The influence of the gut microbiota amyloid formation on the neurodegeneration development was first proposed in 2016. Chen et al. demonstrated that exposing aged rats to curli-producing bacteria leads to the increase of α-synuclein deposition in neurons in both brain and gut, which is a feature of neurodegenerative disorders [171]. Cross-seeding is a potential mechanism, by which gut microbiota can trigger the amyloidogenesis of human proteins. The cross-seeding between bacterial and human amyloids has been demonstrated in vitro: curli fibrils cross-seed fibrillation of amyloid-β [172] and Fap amyloids cross-seed α-synuclein aggregation [173]. The occurrence of human amyloids cross-seeding by prokaryotic amyloids in physiological conditions in vivo represents an important subject for investigation. Nevertheless, the indirect mechanisms of metabolic triggering of human amyloid-associated diseases by gut microbiota without any involvement of cross-seeding cannot be excluded [174,175,176].

The role of amyloids in non-pathogen–host interactions has been less investigated for a while. Some recent demonstration of amyloid properties of RopA and RopB proteins of symbiotic root nodule bacterium *Rhizobium leguminosarum* bv. *viciae* [40] suggests that the bacterial amyloids can be involved not only in the interactions between pathogenic bacteria and multicellular hosts but also in the symbiotic (Type III) interactions with them. Notably, the virulence mechanisms—the ability of a microorganism to successfully infect and colonize host—share similarities between symbiotic bacteria belonging to the order *Rhizobiales* and pathogenic microorganisms [177]. In both cases, adhesion and host tissue surface colonization by bacteria are the key steps in the establishment of the interspecies interaction. In the interaction between pathogenic bacteria and mammalian tissues, bacterial amyloids act as the virulence factors promoting adhesion [178]. The role of amyloids in adhesion of *Escherichia* and *Bacillus* species to plant leaves and roots has also been shown [179,180]. Rhizobia attachment to plant roots is mediated by glucomannans that bind plant lectins in acidic conditions and the synthesis of lipopolysaccharides and cellulose is necessary. Notably, these molecules are a part of extracellular polymeric substances of biofilms [181,182] among them prokaryotic amyloids are widely spread at least in pathogenic species.

Quorum sensing is another mechanism that controls virulence of both pathogenic and symbiotic bacteria [177]. The quorum sensing is a process of gene expression regulation in response to changes in microorganism’s population density. It is mediated by small signal molecules called autoinducers [183] and regulates the transition from free-living form to associated with the multicellular host, modulated adhesion to substrates, and biofilm formation in both pathogenic and symbiotic bacteria [177]. The Fap protein of species from *Pseudomonas* genus, which includes pathogens of plants and animals, forms amyloid fibrils, which transiently bind autoinducers [177]. Thus, prokaryotic amyloids can act as a reservoir of signal molecules and modulate the response of the microbial community to fluctuations in conditions [35].

At the latter stages of chronic infection, both rhizobia and plant pathogens need to avoid or take under control plant defense response. Rhizobia have both specific [184,185] and general, found in rhizobia and phytopathogens, systems to overcome plant immune response such as Type IV and III secretion systems, producing effector molecules to a plant cell [186]. The Type III secretion system of *Rhizobium* is associated with host specificity regulation [187]. In phytopathogens, protein secretion via Type III secretion system promotes resistance to plant immunity and modulates the physiological condition of plant cells for the chronic infection persistence [188]. Type III secretion system effector molecules not only inhibit the plant defense response but can also trigger the hypersensitive response. This group of effector molecules includes harpins, which are secreted proteins of *Xantomonas*, form amyloid fibrils, and elicit the hypersensitive response [43]. Thus, the molecular systems providing virulence of symbiotic and pathogenic bacteria exhibit significant similarity and though amyloids of symbiotic prokaryotes identified to date could probably function as adhesins. Therefore, the real number of amyloids of symbiotic prokaryotes is likely to be significantly higher and may include biofilm scaffold proteins, adhesins, and host immune response modulators. In particular, it has been predicted that proteins bearing potentially amyloidogenic regions are widespread within order *Rhizobiales* that includes both pathogenic and symbiotic species. These proteins are involved in the transport of siderophores and lipopolysaccharides or act as adhesins or flagellum assembly components and contain domains typical for virulence factors [189].

## 8. Conclusions

Prokaryotic amyloids identified to date contribute to various interspecies interactions, including Type I interactions between pathogenic bacteria and multicellular hosts, Type II interactions between different microbial species in the communities, and Type III host–symbiont interactions (the data are summarized in Table 1 and Figure 1). The impact of microbial amyloids in the pathogenesis seems to be significantly underestimated both from the point of view of amyloid biodiversity (numerous novel bacterial amyloids may potentially be found among biofilm-forming pathogenic bacteria species) and the unusual mechanisms of their actions (as in the recently described case of triggering human amyloid diseases by gut microbiota). Even though very few amyloids of symbiotic bacteria have been identified to date, they most likely represent only “the tip of the iceberg” considering the similarity between the molecular systems underlying host–pathogen and host–symbiont interactions including virulence factors to which most prokaryotic amyloids belong. Finally, microbial communities may also be considered as the reservoirs of prokaryotic amyloids involved in both pathogenic and symbiotic interspecies interactions.

## Figures and Tables

**Table 1:** Amyloidogenic proteins of prokaryotes, their properties, functions, and involvement in the interspecies interactions.

Species	Protein	Function of Soluble Protein	Function of Amyloid	Amyloid Properties *		Type of Inter-Species Interactions Mediated by Amyloid **	References
				In Vitro	In Vivo		
Domain: *Bacteria*							
Phylum: *Proteobacteria*							
*Escherichia coli,* *Salmonella enterica*	CsgA (curli), AgfA (tafi)	No data	Biofilm matrix protein; surface adhesion; intercellular adhesion	CR ( Congo red) absorbance, ThT (Thioflavin T) fluorescence, CD (Circular dichroism), FTIR (Fourier-transform infrared spectroscopy), XDR (X-ray diffraction)	Extracellular fibrils formation	I	[29,30,31,32]
*Pseudomonas aeruginosa,* *P. fluorescens,* *P. putida*	FapC	No data	Biofilm matrix protein; facilitates mechanical stiffness; enhances hydrophobic properties; binds quorum-sensing signal molecules	TEM (Transmission electron microscopy), FTIR, XDR	Extracellular fibrils formation; purified native fibrils: CD, FTIR, ThT fluorescence	I	[33,34,35]
*Legionella pneumophila*	Not identified	No data	Biofilm matrix protein	No data	ThT fluorescence, CR staining and WO1 antibodies binding of extracellular polymer matrix of biofilm	I	[36]
*Escherichia coli*	OmpA	Outer membrane porin	Virulence factor; amyloid function is unknown	ThT fluorescence, TEM, CD (for N-terminal domain)	No data	I ***	[37]
*Escherichia coli*	OmpC	Outer membrane porin	Virulence factor; amyloid function is unknown	Proteinase K resistance, TEM, ThT fluorescence, CR absorbance and birefringence	No data	I ***	[38]
*Mannheimia haemolytica*	OmpP2-like protein	Outer membrane porin	Biofilm matrix protein; adhesion to host’s tissues	CR binding	Fibrils on the cell surface, binding anti-OmpP2-like protein antibodies	I	[39]
*Rhizobium leguminosarum*	RopA	Outer membrane porin	Component of extracellular capsule	CD, CR birefringence, ThT fluorescence, TEM, detergent-resistance, trypsin resistance	SDS (Sodium dodecyl sulfate)-resistant polymer formation, fibrils on the cell surface, binding anti-RopA antibodies	III ***	[40]
*Rhizobium leguminosarum*	RopB	No data	Component of extracellular capsule	CD, CR birefringence, ThT fluorescence, TEM, detergent-resistance, trypsin and pepsin resistance	SDS-resistant polymer formation, fibrils on the cell surface, binding anti-RopB antibodies	III ***	[40]
*Klebsiella pneumoniae*	Microcin E492	Pore-forming toxin	Toxin inactivation	TEM, CD, ThT fluorescence, CR absorbance, proteinase K resistance, XDR	Fibril formation on the surface of Microcin E492 secreting strain (TEM)	II	[41,42]
*Xanthomonas axonopodis*	HpaG (harpin)	No data	Virulence factor; induces plant hypersensitive response	TEM, CD, CR absorbance and birefringence, proteinase K resistance	No data	I ***	[43]
*Pseudomonas syringae*	HrpZ (harpin)	No data	Virulence factor; induces plant hypersensitive response	TEM	No data	I ***	[43]
*Erwinia amylovora*	HrpN (harpin)	No data	Virulence factor; induces plant hypersensitive response	TEM	No data	I ***	[43]
*Gallibacterium anatis*	EF-Tu	Elongation factor	Biofilm matrix protein; surface adhesion	TEM	TEM, CR binding, antibodies against curli	N/a	[44]
Phylum: *Firmicutes*							
*Bacillus subtilis,* *Bacillus cereus*	TasA	No data	Biofilm matrix protein; facilitates biofilm integrity; binds exopolysaccharides on the initial steps of multispecies biofilm formation	TEM, CD, NMR (Nuclear magnetic resonance), FTIR	Anti-TasA antibodies binding extracellular fibrils in biofilm matrix; native fibrils: TEM, CR absorbance, ThT fluorescence	I, II	[45,46,47]
*Staphylococcus aureus*	PSMs	No data	Biofilm matrix protein	TEM, ThT fluorescence, NMR (cross-α structure)	Extracellular fibrils in biofilm matrix while *Δabpsm* mutant were unable to form fibrils	I	[8,48]
*Staphylococcus aureus*	SuhB	No data	Biofilm matrix protein; intercellular adhesion	CR absorbance, ThT fluorescence, FTIR, SEM, XDR	No data	I ***	[49]
*Staphylococcus aureus*	AgrD	Propeptide; autoinducing peptide pheromone (AIP) precursor	*N*-terminal peptide, cleaved during AIP maturation, forms amyloid; biofilm matrix component	*N*-terminal domain: ThT fluorescence, TEM, CR absorbance, CD	Fibrils formed by *N*-terminal domain of AgrD in biofilm matrix	I	[50]
*Staphylococcus aureus*	Bap	No data	Surface adhesion; intercellular adhesion; promotes biofilm formation in acidic conditions	Bap B-domain: ThT fluorescence, CR absorbance, TEM, FTIR, CD	Anti-Bap antibodies binding fibrils formation on the cell surface	I	[51]
*Enterococcus faecalis*	cOB1	Pheromone; part of the pheromone-based conjugation system	Prevention of conjugation; initiate the aggregation of biofilm matrix proteins (such as Esp)	ThT fluorescence, CR absorbance, CD, TEM	No data	II ***	[52]
*Enterococcus faecalis*	Esp	No data	Biofilm matrix protein	C-DAG assay: CR binding, TEM; *N*-terminal domain: SEM (Scanning electron microscopy), FTIR, CD, CR absorbance, ThT fluorescence; Fibril formation on the surface of Δ*bap S. aureus* expressing Esp_N	No data	I ***	[53]
*Staphylococcus epidermidis*	Aap	Intercellular adhesion	Biofilm matrix protein	ThT fluorescence, CR absorbance, TEM, CD	SDS-resistant aggregates, binding anti-Aap antibodies, were extracted from biofilm-forming bacteria	I	[54]
*Staphylococcus epidermidis*	Sbp	No data	Scaffolding protein in biofilms	TEM, AFM, FTIR, CR absorbance, ThT fluoerescence	ThS-binding inclusions, expressing Sbp	I	[55]
*Streptococcus mutans*	Adhesin P1	No data	Biofilm matrix protein; adhesion to tooth surface	CR birefringence, ThT fluorescence, TEM, XDR	No data	I ***	[56]
*Streptococcus mutans*	WapA	No data	Biofilm matrix protein	CR birefringence, ThT fluorescence, TEM, XDR	No data	I ***	[57,58]
*Streptococcus mutans*	Smu_63c	No data	Biofilm matrix protein	CR birefringence, ThT fluorescence, TEM, XDR	No data	I ***	[57,58]
*Bacillus subtilis*	HelD	Helicase	Amyloid function is unknown	CD, ThT fluorescence, CR absorbance, XDR	ThS-binding inclusions in strain, overexpressing HelD	N/a	[59]
*Clostridium botulinum*	Rho ****	Transcription terminator	Modulates transcription; causes genome-wide changes in transcriptome	Analysis of prion-like domain: ThT, ThS and CR fluorescence, FTIR, TEM; C-DAG (Curli-dependent amyloid generator) assay: CR birefringence, SDS-resistance	SDS-stable aggregate formation in *E. coli*	N/a	[60,61]
*Solibacillus silvestris*	Bioemulsifier BE-AM1	No data	Cell surface properties modulation; biofilm matrix protein	CR birefringence, FTIR, CD, TEM	No data	N/a	[62,63]
*Listeria monocytogenes*	Listeriolysin O	Toxin, that forms pores in phagolysosome’s membrane	Toxin inactivation	CD, TEM, CR fluorescence and absorbance, ThT fluorescence, trypsin resistance	No data	I	[64]
Phylum: *Actinobacteria*							
*Streptomyces coelicolor*	ChpA-H (chaplin)	No data	Lowering of the surface tension; assists aerial hyphae formation	CD, TEM, XDR, FTIR	Native extracts: ThT fluorescence, TEM, CD	N/a	[65,66]
*Streptomyces coelicolor*	RdlB (rodlin)	No data	Rodlet layer formation; assists aerial hyphae formation	ThT fluorescence, TEM, CD, XDR	No data	N/a	[67]
*Mycobacterium tuberculosis*	CarD	Transcription factor	Amyloid function is unknown	ThT fluorescence, TEM, SDS-resistance, CD (increase in β-sheet content during heating)	ThS-binding (Thioflavin S) inclusions in strain, overexpressing CarD	N/a	[68]
*Mycobacterium tuberculosis*	MTP	No data	Adhesion to host’s tissues	TEM, CR binding	TEM, SDS resistance of fibrils	I ***	[69]
Domain: *Archaea* Phylum: *Euryarchaeota*							
*Haloferax volcanii*	Not identified	No data	Biofilm matrix protein	No data	Fluorescence of biofilms stained with CR and ThT	N/a	[70]
*Methanosaeta thermophila*	MspA	No data	Tubular sheaths component; facilitates its stiffness	TEM, ThT, CD, FTIR, XDR	Intact sheaths: TEM, WO1 antibodies; purified sheaths: TEM, WO1 antibodies, ThT, FTIR, XDR	N/a	[71]
*Methanospirillum hungatei*	MspA	No data	Tubular sheaths component; facilitates its stiffness	No data	Intact sheaths: WO1 antibodies; purified sheaths: TEM, FTIR	N/a	[72]

* CR, Congo red; ThT, Thioflavin T; ThS, Thioflavin S; C-DAG, Curli-dependent amyloid generator; CD, Circular dichroism; FTIR, Fourier-transform infrared spectroscopy; XDR, X-ray diffraction; NMR, Nuclear magnetic resonance; SDS, Sodium dodecyl sulfate; SEM, Scanning electron microscopy; TEM, Transmission electron microscopy. ** Type of proven or hypothetical inter-species interactions: Type I, host–pathogen interactions; Type II, interactions between different microbial species in the communities; Type III, host–symbiont interactions; N/a, not applicable. *** Hypothetical interaction based on the structural protein function. **** This protein also possesses infectious prion properties [60].

## Abbreviations

AIP	Autoinducing peptide
CD	Circular dichroism
C-DAG	Curli-dependent amyloid generator
CR	Congo red
EAEC	Enteroaggregative *Escherichia coli*
eDNA	Extracellular DNA
EPS	Extracellular polymeric substances
FTIR	Fourier-transform infrared spectroscopy
LLO	Listeriolysin O
NMR	Nuclear magnetic resonance
NMR	Nuclear magnetic resonance
PSMα3	Phenol-soluble modulin α3
SDS	Sodium dodecyl sulfate
SEM	Scanning electron microscopy
TEM	Transmission electron microscopy
ThS	Thioflavin S
ThT	Thioflavin T
XRD	X-ray diffraction
